# Mechanisms and Neuroimaging Patterns of Hypereosinophilia-Related Ischemic Stroke: A Narrative Review through Three Cases

**DOI:** 10.3390/jcm11195595

**Published:** 2022-09-23

**Authors:** Maria Cristina Cioclu, Francesco Cavallieri, Manuela Napoli, Claudio Moratti, Rosario Pascarella, Franco Valzania, Marialuisa Zedde

**Affiliations:** 1 Neurology Unit, OCB Hospital, Azienda Ospedaliera-Universitaria, 42123 Modena, Italy; 2 Neurology Unit, Neuromotor and Rehabilitation Department, Azienda USL-IRCCS di Reggio Emilia, 42124 Reggio Emilia, Italy; 3 Neuroradiology Unit, Radiology Department, Azienda USL-IRCCS di Reggio Emilia, 42124 Reggio Emilia, Italy

**Keywords:** stroke, hypereosinophilia, hypereosinophilic syndrome (HES), brain MRI, embolic pattern, border zone stroke

## Abstract

Background: Hypereosinophilic syndromes (HES) are a group of relatively rare disorders in which neurological manifestations, including ischemic stroke, are common. The hypothesized pathophysiological mechanisms are hypercoagulability, cardioembolism (mainly mediated by myocardial involvement) and damage to the endothelium. A variable ischemic pattern has been described, including an association of territorial and border zone ischemic stroke. Methods: Three patients who presented to our department with acute stroke were selected aiming to show these three different mechanisms inferred from the stroke pattern on brain Magnetic Resonance Imaging (MRI) and to simultaneously illustrate the three main causes of HES. Results and Discussion: The first patient is a 55-year-old man with an abrupt onset of aphasia due to an acute ischemic stroke involving the left parietal lobule and the angular gyrus; recent lab test had shown hypereosinophilia. An extensive workup excluded primary and secondary causes of hypereosinophilia so a diagnosis of idiopathic hypereosinophilia was done and he was treated with high doses of steroids. The second patient had severe hypereosinophilia and developed multiple small, scattered ischemic lesions, mainly in border zone zones. The history of severe asthma and recurrent sinusitis supported the diagnosis of EGPA (Eosinophilic Granulomatosis with Polyangiitis); considering the severe clinical conditions and the presumptive role of hypereosinophilia in determining her symptoms, steroid treatment was promptly started, with good clinical response. The third patient also presented with multiple metachronous ischemic lesions, both in cortical and border zone distribution and marked eosinophilia; the diagnostic work-up found an ovarian cancer. She was treated with steroids and then underwent surgery and adjuvant chemotherapy. Conclusions: HES should be considered in stroke etiological evaluation, although it is a rare disorder, and border zones pattern without large artery steno-occlusion on neuroimaging may help to raise the suspicion in the neurovascular diagnostic pathway. A thorough research of the sources of hypereosinophilia should be performed to select the appropriate therapy.

## 1. Background

According to the most recent classification criteria [1], hypereosinophilic syndromes (HES) are a group of disorders characterized by the presence of hypereosinophilia associated with eosinophil-mediated organ damage. Hypereosinophilia is defined as an elevation in the absolute eosinophil count (>1.5 × 10^9^/L or >1500 cells/microL) in the peripheral blood in two distinct determinations, performed at an interval of at least one month, and/or tissue hypereosinophilia (more than 20% of eosinophils in bone marrow sections, and/or extensive tissue infiltration by eosinophils, and/or marked deposition of eosinophils granule proteins). The one-month interval should not be considered when the symptoms of eosinophil-mediated organ dysfunction require immediate therapy [1].

On the basis of the pathogenic mechanism underlying the increased production of eosinophils, HES can be further classified as:

– primary or neoplastic HES,
– secondary or reactive HES,
– idiopathic HES (when the other causes have been excluded) and
– other conditions and syndromes accompanied by hypereosinophilia.

In primary HES the most involved neoplasms are myeloid neoplasms: chronic myeloid leukemia, other myeloproliferative neoplasms (MPN), distinct variants of acute myeloid leukemia, rare forms of myelodysplastic syndromes (MDSs), some MDS/MPN overlap disorders, and a subset of patients with (advanced) systemic mastocytosis (SM) [1].

Reactive or secondary HES is associated with an underlying inflammatory condition (e.g., inflammatory bowel diseases) or solid tumors, but also some hematological neoplasms (Hodgkin lymphoma, T-cell lymphoma, or B-lymphoblastic leukemia/lymphoma). Other subsets of reactive HES is related to helminth infections, allergic reactions, atopic diseases and drug reactions.

The third subtype includes eosinophilic granulomatosis and polyangiitis (EGPA, formerly known as Churg Strauss Syndrome) or other organ specific conditions (for example chronic eosinophilic pneumonia and others) [1]. HES are rare, although there are few data concerning the epidemiology; using the data from the Surveillance, Epidemiology and End Results (SEER) database from 2001–2005 in the US, an age-adjusted incidence rate of approximately 0.036 per 100,000 was calculated [2].

Neurologic involvement is not uncommon (65% of cases) in HES and it typically consists in peripheral neuropathy, encephalopathy, and stroke [3]. More in detail, 15% of patients with HES have central nervous system involvement, mainly as an encephalopathy characterized by behavioral disturbances and upper motor neuron signs, but 12% of patients have cerebrovascular events with an embolic pattern; 52% of patients have a peripheral neuropathy, more commonly a sensory polyneuropathy [3]. Previous reports of stroke in the context of HES involved either large vessel occlusion (or territorial) pattern and border zone pattern; the pathophysiological mechanisms, even if not completely understood, might be related to hypercoagulable state, probably due to the cytotoxic effect of eosinophil-produced mediators and subsequent endothelial damage and thromboembolism caused by cardiac involvement [4,5,6,7,8,9]. In this setting we report three cases of ischemic lesions as the first main clinical expression of different types of HES.

## 2. Methods

Among patients admitted to a single center Stroke Unit in the last 5 years, we selected the unique three cases of stroke as presenting manifestation of HES. The patients underwent a complete diagnostic work-up in order to exclude other causes of stroke and to ascertain the cause of HES. All patients underwent a hematological consultation because of HES and the exclusion of hematological malignancies was carried out accordingly to the current guidelines. A detailed neuroimaging study, including Computed Tomography Angiography (CTA) and brain MRI with Magnetic Resonance Angiography (MRA) was performed in our institution as well as the screening for systemic involvement though whole body Computed Tomography (CT) and cardiological tests (including prolonged heart rhythm monitoring and echocardiography) were performed as part of the routine diagnostic pathway of patients with ischemic stroke at our institution.

## 3. Results

### 3.1. First Case

**Figure 1:**
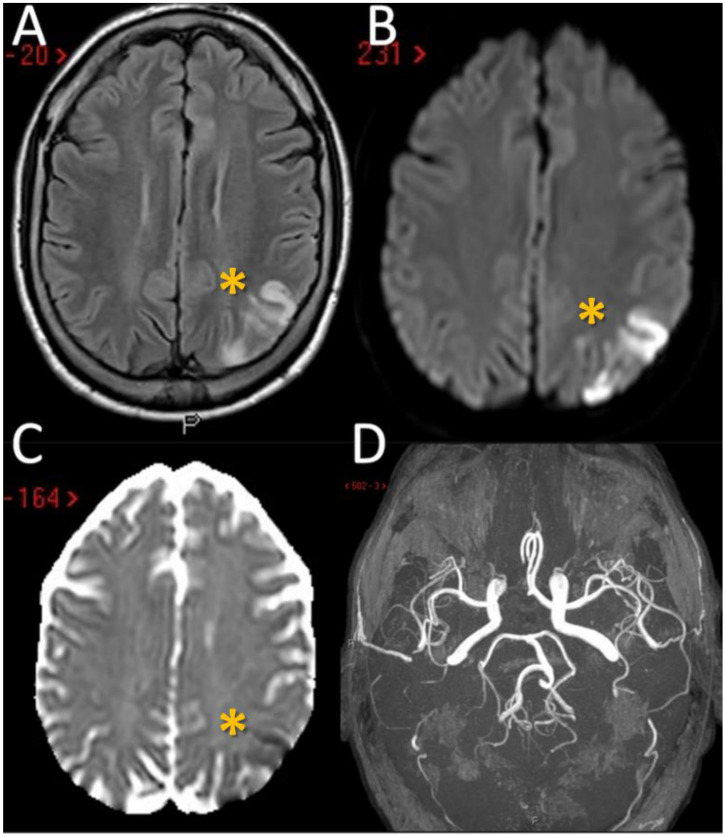
Brain MRI of the first patient. MRI of patient 1 showed the presence in the axial Fluid Attenuated Inversion Recovery (FLAIR) sequences of a wedge of cortical ischemic lesions in the left posterior parietal lobe (yellow asterisk) (**A**) with DWI and ADC sequences pattern suggesting acute ischemic lesions (**B**,**C**). MRA (time-of-flight [TOF] reconstruction) shows fully patent proximal intracranial arteries and right A1 anterior cerebral artery (ACA) aplasia (**D**). MRI: Magnetic Resonance Imaging; MRA: Magnetic Resonance Angiography; DWI: Diffusion Weighted; ADC: Apparent Diffusion Coefficient.

The first patient is a 55-year-old man who was first admitted to the Emergency Department after the acute onset of confusion, altered speech comprehension and production. The episode lasted 45 min and spontaneously resolved. Head CT and neurosonological examination of the neck and cerebral vessels were unremarkable, and the patient was discharged on single antiplatelet therapy (acetylsalicilic acid) with a diagnosis of transient ischemic attack (TIA). He went back to the hospital two days later after developing writing difficulties, and he was admitted to our Stroke Unit; no further focal neurological deficits were evident on clinical examination. The most relevant aspects of his past medical history were mild hyperlipidaemia and smoking; recent lab tests performed before admission revealed an increase in the absolute and relative eosinophil count above the threshold for HES diagnosis. A second head CT at two days from the symptom onset showed an ischemic lesion involving the left parietal lobule and angular gyrus, confirmed by a Magnetic Resonance Imaging (MRI) scan (Figure 1A–D).

An extra- and intracranial CTA was normal as well as a transthoracic echocardiography and an electrocardiogram (ECG)-Holter; no right-left shunt was found on transcranial Doppler ultrasonography (TCD); thrombophilia screening and tests for autoimmunity were unremarkable. On lab examination a moderate hypereosinophilia was confirmed (absolute eosinophil count of 2290/microL). An extensive screening for primary and secondary causes of hypereosinophilia was performed including a total body CT, stool samples for parasites, tryptase and B12 levels determination, hematologic evaluation, lymphocyte typing, bone marrow biopsy and testing for Fip1-like 1 (FIP1L1) and platelet-derived growth factor receptor alpha (PDGFRA) fusion gene, which were all negative. The patient reported an episode of pruritus occurred two months before hospitalization, responsive to steroid treatment. He underwent allergic tests that were positive for Balsam of Peru (BP) and bet v1. Although an atopic condition, which is a known cause of eosinophilia, was identified, it appears unlikely that the persistent hypereosinophilia with eosinophil counts >1500/microL in our patient could be explained by allergy alone (4,10). Moreover, BP is one of the most common contact allergens and reactions to BP are typically seen as contact eczema or dermatitis both for topical contact and oral intake with several systemic complications and the reported patient did not have any of these manifestations. Therefore, a diagnosis of idiopathic hypereosinophilia was done.

The patient was treated with high-dose intravenous glucocorticoid therapy (methylprednisolone 1000 mg each day for five days, followed by slow tapering off) and discharged on oral prednisone 25 mg, with a significant reduction in eosinophil count in the peripheral blood and clinical improvement.

### 3.2. Second Case

**Figure 2:**
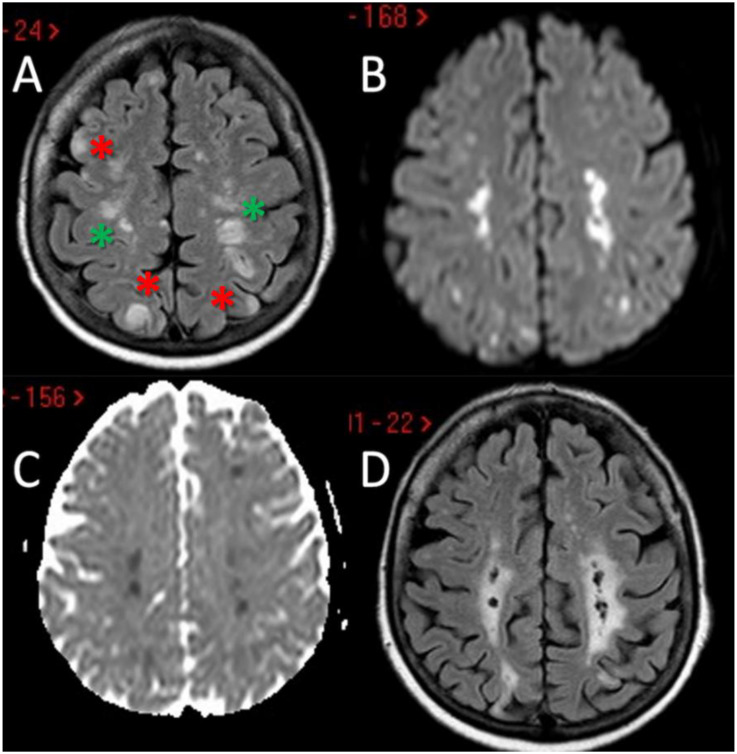
Brain MRI of the second patient. MRI of patient 2 showed the presence of multiple scattered hyperintense brain lesions on both internal border zone (green asterisk) and on cortical location (red asterisk) at axial FLAIR sequences (**A**) with diffusion weighted imaging (DWI) and apparent diffusion coefficient (ADC) pattern suggesting acute ischemic lesions with a metachronous timing pattern (**B**,**C**). In the follow-up MRI performed 3 months later it is evident the chronical evolution of the above-mentioned ischemic lesions (**D**).

The second patient is a 64-year-old woman with a previous history of arterial hypertension and paroxysmal atrial fibrillation, on antiplatelet treatment with aspirin and rhythm control with flecainide; she also reported asthma and recurrent sinusitis (a prior sinus CT had displayed severe inflammation of the paranasal sinuses). She arrived at our attention because of bilateral upper limbs paraesthesia followed, the next day, by psychomotor slowing, right upper limb weakness, instability, and difficulty with walking. Neuroimaging exams (head CT and MRI) revealed multiple bilateral cortical-subcortical ischemic lesions in the cerebral and cerebellar hemispheres, mostly located in border zone areas (Figure 2A–D); no stenosis or occlusion in the head and neck vessels was detected on CTA and MRA.

Neurological exam was significant for severe right upper limb paresis and mild left ataxic hemiparesis. The echocardiogram showed akinesia of the apical portion of the left ventricle, without evidence of intracardiac thrombi and a stress cardiomyopathy was suspected. Apixaban was prescribed considering the previous finding of atrial fibrillation. She presented a marked hypereosinophilia in the peripheral blood (5540/microL) and therefore a thorough evaluation of the possible causes was performed: neoplastic markers, parasite tests, serological tests for HIV, Mycoplasma, Borrelia, Syphilis, Vitamin B12, tryptase as well as a blood smear and lymphocyte phenotyping on peripheral blood were all within normal range. The bone marrow biopsy showed increased cellularity and increased eosinophils so cytogenetic and molecular studies including BCR/ABL1, PDGFRA, PDGFRB, FGFR1, PCM1-JAK2 fusion genes were performed and resulted negative. A positive high titer ANA- and AMA-test was detected (1:1280) whereas ANCA test was negative. The whole-body CT scan showed areas of subpleural ground glass in the superior lobe of the left lung, splenic infarcts, and enlargement of para-esophageal and mediastinal lymph nodes. The main findings of the whole body CT scan are the lack of solid neoplasms and the evidence of splenic infarcts. Both lungs and lymph nodes changes have been considered not specific in a bedridden patient with delirium. Heart MRI was then performed which displayed no abnormalities. During the hospitalization, the patient’s conditions worsened, and she developed quadriparesis, altered mental status, frequent episodes of delirium and hallucinations that required pharmacological sedation. Therefore, in the hypothesis of stroke and encephalopathy in the context of a HES (possibly EGPA, although it was impossible to perform a biopsy because of the severe clinical conditions and no surrogate for vasculitis was found—neurophysiologic studies were also not performed because of the severity of her conditions and also because they wouldn’t have changed the patient acute management), the patient was started on high doses of intravenous corticosteroids (1 g/day for 5 days), followed by oral tapering. The treatment determined a marked clinical improvement and a reduction in the number of eosinophils; following rheumatologic evaluation, during steroid tapering, the patient was started on cyclophosphamide (i.v. treatment, 0.5–1 g/m^2^ for 6 months, according to the regimen advised by American National Institute of Health). She was then referred to a rehabilitation center and her conditions continued to improve both on the cognitive and on the motor level. After the cyclophosphamide treatment, a maintenance therapy first with azathioprine and then, for side effects (liver enzymes elevation), with methotrexate was started. The patient continues rheumatologic, pneumological and neurologic follow-up.

### 3.3. Third Case

**Figure 3:**
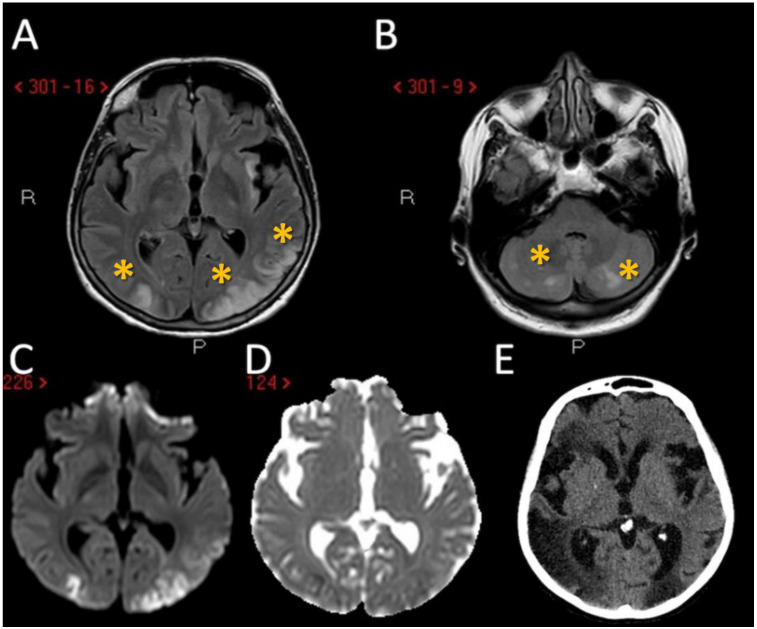
Neuroimaging studies of the third patient. MRI of patient 3 showed the presence of multiple supratentorial (**A**) and infratentorial (**B**) hyperintense lesions (yellow asterisk) with a territorial distribution in A and border zone pattern in B (axial FLAIR sequences) with DWI and ADC pattern suggesting acute ischemic lesions with a metachronous timing pattern (**C**,**D**). In the follow-up CT performed 7 days later it is evident the increased number of ischemic lesions, in particular on the right hemisphere (**E**).

The third patient is a 69-year-old woman with a remote history of herpetic encephalitis and transient global amnesia who arrived at the Emergency Room after developing low grade fever and bilateral visual blurring. Multiple synchronous ischemic lesions, located bilaterally, both in infra- and supra-tentorial regions, mainly in border zones, with a prevalent involvement of the occipital lobes were evident on neuroimaging without large vessel occlusion or stenosis were found (Figure 3A–E).

Transthoracic echocardiography did not find significant abnormalities and, in particular, heart valves were functioning and without vegetations. Laboratory examination showed a CBC of 11.700 cell/microL with 11% eosinophils (1280 cells/microL) and a very high D-dimer level (>35,000). Although her eosinophil count was a little less than 1500, not strictly meeting the criteria for hypereosinophilia, considering the significantly increased value and the co-occurrence of a very high D-dimer, a steroid treatment was started and an extensive diagnostic workup was performed, showing an increased level of CA125 (189.3 U/L); whole-body CT scan revealed a right ovarian mass of 6 cm of diameter, besides splenic and renal infarcts. Thoracic CTA was negative for pulmonary embolism. Intravenous high doses of corticosteroids were started soon after admission (1 g of methylprednisolone for 3 days followed by 500 mg for 2 days) with subsequent oral tapering with some degree of clinical improvement and eosinophil count normalization. She later underwent laparoscopic peritoneal washing and adnexectomy and laparotomic total hysterectomy with partial peritonectomy. The histopathologic diagnosis was clear-cell ovarian carcinoma (pTNM: pT3a, Nx); analysis of the BRCA1 and 2 mutation was negative. After surgery 6 cycles of chemotherapy with paclitaxel and carboplatin were administered and at a one-year-follow up there are no clinical or radiological signs of relapse. However, she presented a progressive decline in her cognitive performances with behavioral changes and, more recently, also in her motor abilities.

The main clinical features of the three cases are reported in Table 1.

## 4. Discussion

**Figure 4:**
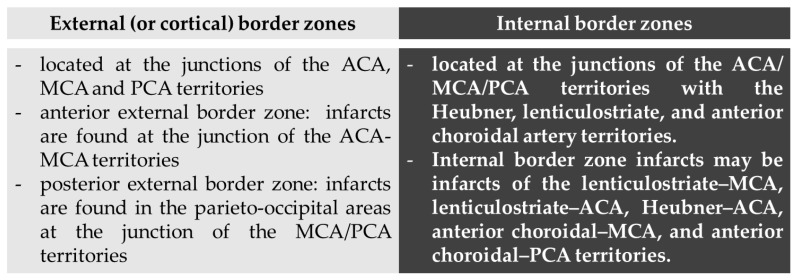
Types of border zones and corresponding infarcts according to their location. Abbreviations: MCA: middle cerebral artery; ACA: anterior cerebral artery; PCA: posterior cerebral artery.

We report three patients with stroke as presenting symptom of hypereosinophilia from three different causes, two of them fulfilling the criteria for HES [1]. The pathophysiological mechanisms of stroke in the context of hypereosinophilia may produce a composite pattern on neuroimaging studies, ranging from a large vessel occlusion (or territorial) pattern [8] to multiple scattered lesions in several different vascular territories, and in particular in border zones [5,6,9,10]. These last ones may be considered a hallmark of HES-related stroke, in particular without evidence of hemodynamic unfavorable conditions, being caused by a direct toxic effect of the increased number of circulating eosinophils to the endothelium of small cerebral vessels. Border zone infarcts are ischemic lesions that are localized at the borders of the distal fields of two non-anastomosing arterial systems [11,12,13]. These lesions are approximately 10% of all brain infarcts and, although their pathophysiology is yet a matter of discussion, the more accepted hypothesis is that decreased (or misery) perfusion in the distal regions of the vascular territories leaves them vulnerable to infarction because of the end-type arterioles without an efficient anastomotic network among superficial and deep territories (e.g., in middle cerebral artery—MCA—supply) or MCA to posterior cerebral artery/anterior cerebral artery (PCA/ACA) and PCA to ACA borders [13,14], as demonstrated in an autoptic study [15]. Traditionally, two types of border zone infarcts are identifiable according to their location: external (or cortical) and internal (or subcortical) type [13], as detailed in Figure 4.

**Figure 5:**
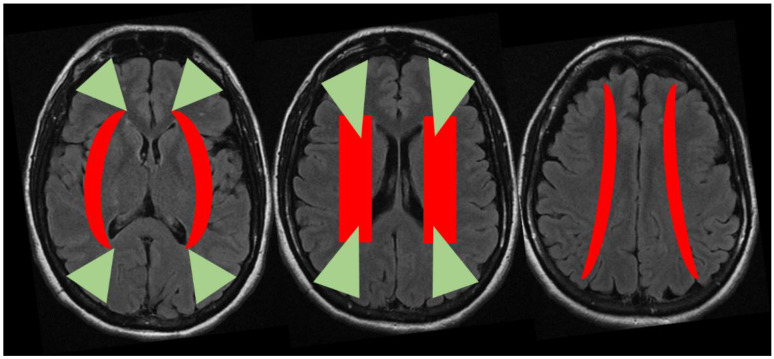
Putative location of the external (green) and internal (red) border zones superimposed on three sequential axial slices of brain MRI (FLAIR sequence). Abbreviations: FLAIR: Fluid Attenuated Inversion Recovery.

The anterior external border zone infarcts are more common than posterior ones because of the high prevalence of ICA disease. Indeed, posterior external border zones infarctions require vertebrobasilar disease with superimposed fetal-type PCA. Among internal border zones infarcts the most common location is on the lenticulostriate–MCA border zone, supplied by the end branches of deep perforating lenticulostriate arteries and medullary penetrators from the pial–MCA. The putative external and internal border zones are superimposed on axial brain MRI images in the Figure 5. However, it is important to consider that the extension and localization of the border zones can undergo variations in single individuals both in normal conditions and in situations that create chronic hemodynamic impairment. Moreover, the anatomical and functional organization of white matter vasculature may take account of different patterns of border zones, accordingly to a centripetal/centrifugal or interconnected organization [16].

The main pathophysiological hypothesis about border zone infarcts is the hemodynamic impairment downstream a severe arterial stenosis or occlusion with basal low perfusion pressure which made these regions more susceptible to ischemia and finally infarction. Another opposite view is that there is an association between border zone infarction and microemboli, supported by autoptic studies [17]. One of the hypotheses unifying these two theories, specifically raised in the setting of border zone infarctions, is that the clearance of emboli and microemboli is impaired in regions with hemodynamic impairment [18,19] and this hypothesis has been supported by experimental data [20].

Another relevant issue is that external and internal border zone infarcts may have a different pathogenesis, which may be inferred by neuroimaging appearance. As shown in Figure 5 (green triangles), external border zone infarcts usually are wedge-shaped and their hemodynamic origin is hard to define only on the neuroimaging basis because of the wide variability of arterial territories and leptomeningeal collaterals. Therefore, isolated external border zone infarctions may be embolic rather than purely hemodynamic and they may be associated to small cortical infarcts. The cause of unilateral posterior external border zone infarcts is more often embolic than hemodynamic; on the contrary, bilateral infarcts are more likely to be caused by underlying hemodynamic impairment [21]. Instead, internal border zone infarcts are usually multiple and occur in a rosary-like pattern (as seen in Figure 2 for the second patient we described). A unique widely shared definition of this pattern does not exist; it has been assumed from one of the first reports that the rosary-like pattern is a series of three or more lesions, each with a diameter of 3 mm or more, arranged in a linear pattern parallel to the lateral ventricle in the centrum semiovale or corona radiata [22]. Internal border zone infarcts are also categorized as partial or confluent [12]; partial infarcts are large, elongated (cigar shaped), and arranged in a line parallel and adjacent to the lateral ventricle, looking like the beads of a rosary. It has been postulated that the duration of hemodynamic impairment is related to a partial o confluent appearance, respectively with a shorter and longer lasting impairment [23]. The main neuroimaging differential diagnosis of internal border zone infarcts is represented by superficial perforator (medullary) infarcts, caused by caused by the occlusion of medullary arteries arising from pial plexuses, because of the similar appearance on MRI. Usually they are smaller, more superficially located and widely scattered than internal border zone infarcts and they have a tendence to spare the paraventricular regions, more typically affected in internal border zone infarcts. The hemodynamic factor is much more evident in internal border zone infarcts than in external border zone infarcts, mainly because the greater vulnerability of the internal border zone due to anatomical feature of the supplying arterioles. Indeed, internal border zones are supplied by medullary penetrating vessels branching from MCA and ACA and by deep perforating lenticulostriate branches. The first ones are the most distal internal carotid artery (ICA) branches with the lowest perfusion pressure. The second ones have poor collateral supply, and unfortunately, there are no anastomoses between the deep perforators and the white matter medullary arterioles [16]. The consequence of this anatomical organization of the cerebral vasculature is that the centrum semiovale and corona radiata are more susceptible than other brain regions to hemodynamic-related ischemia. Conversely, the external border zone is closer to the cortical surface, where shorter penetrating arteries originate, and it has a better chance of developing a collateral supply through leptomeningeal or dural anastomoses [13,16]. The higher probability of hemodynamic impairment may be predicted when external and internal border zone infarctions co-occur. Although more known in the supratentorial compartments, there is a corresponding border zones organization in the cerebellum. Border zone infarcts in the cerebellum are usually less than 2 cm in size and are located at the borders of the anterior inferior cerebellar artery (AICA), superior cerebellar artery (SCA), posterior inferior cerebellar artery (PICA), and their branches. The pathophysiology of cerebellar border zones infarctions is similar to the one of supratentorial border zone infarctions, combining the hemodynamic and embolic hypotheses. As expected, small border zone lesions may coexist with large territorial infarctions. The bilateral cerebellar infarcts shown in Figure 3 for the third patient may have a predominantly hemodynamic (border zone between lateral and medial branches of PICA o both sides) than embolic pattern, whereas supratentorial infarctions in the same patient have a clear territorial pattern.

Typically, border zone infarcts have a hemodynamic basis and occur in the context of severe stenosis of extracranial or intracranial arteries, or in systemic conditions that cause brain hypoperfusion such as severe hypotension and cardiac arrest [11,13] but in HES the pathogenesis is different, as detailed below. In particular, it has been reported that, although rare, hypereosinophilia may be complicated by multiple ischemic strokes including a border zones involvement, often bilateral and symmetrical, without upstream stenosis or occlusion, i.e., without a significant hemodynamic factor [24]. This involvement may be in the external and internal border zones, often in association, and it may be accompanied by cortical infarctions. One of the possible causes described in literature is concomitant endomyocardial fibrosis that can be responsible for intracardiac thrombi formation and microembolization [9]; none of our patients showed endomyocardial involvement but heart MRI, which is the non-invasive exam with the best diagnostic yield, was performed in only one of the three patients described. In addition, endothelial damage, caused by the release of cytotoxic substances from eosinophils might play a role in thrombi formation and infarction [25]; high eosinophil count might also be a cause for hyperviscosity and subsequent microcirculation alteration [6]. All these mechanisms may be coexistent and synergistically lead to microemboli and local thrombi formation that might lead to stroke; the preferential involvement of border zone regions seems to be connected to lower washout ability of these areas compared to others in the brain, because of their lower perfusion pressure; this is the so called “impaired wash-out theory” [4,5]. For this reason, although our second patient had also a history of atrial fibrillation, a known cause of multiple ischemic lesions in different vascular territories, the clinical picture, and the distribution of the lesions, mainly in border zone areas, suggested that hypereosinophilia was the most likely underlying cause; she was nonetheless treated for both conditions and behavioral changes were only responsive to steroid therapy. Considering her history of severe asthma and recurrent sinusitis, EGPA was suspected although the patient had no sign of vasculitis, therefore not strictly meeting the diagnostic criteria. Although rare, hypereosinophilia can be paraneoplastic, associated with solid tumors; it was for instance described in lung, gastrointestinal [26,27] and in ovarian cancer [27] as in the case of our third patient. In this scenario it is thought that the tumor cells are responsible for the increased eosinophils production because they produce cytokines that support eosinophil proliferation, growth, and survival [26]. Besides, it is well known that thromboembolism is a frequent paraneoplastic syndrome [28] and, as stated before, hypereosinophilia per se is pro-thrombotic; even if the eosinophil count in our patient was a little under the 1500 cells/microL-cut-off that defines hypereosinophilia we believe that her marked eosinophilia concurred, together with paraneoplastic thrombophilia in determining her cerebral but also her visceral infarcts. The treatment for these paraneoplastic syndromes is the treatment of the malignancy itself. In the first patient described, primary and secondary causes of hypereosinophilia were excluded so a diagnosis of Idiopathic Hypereosinophilic Syndrome was made. The patient responded well to steroids that are considered to be the first-line therapy; in some instances, corticosteroid-sparing agents (hydroxyurea, interferon alpha, or anti-IL-5) could be necessary to prevent relapses in the case of steroid side effects [29,30]. Early initiation of therapy is important since it can prevent disease progression.

## 5. Conclusions

In conclusion, HES should be considered in the diagnostic work-up of ischemic stroke and the suspicion may be raised and supported by a neuroimaging pattern of lesions symmetrically involving the border zones, in the presence of a marked increase in eosinophils’ count. An extensive diagnostic work-up is hence necessary to establish the type of HES and the correct treatment which should be started promptly.

## Figures and Tables

**Table 1:** Main clinical features of the three patients.

Patients	Patient 1	Patient 2	Patient 3
Age (years)	55	64	69
Sex	M	F	F
Cardiovascular risk factors			
Arterial Hypertension	No	Yes	No
Diabetes Mellitus	No	No	No
Hyperlipidemia	Yes	No	No
Atrial Fibrillation	No	Yes	No
Coronary Artery Disease	No	No	No
Smoking	Yes	No	No
Obesity	No	No	No
Previous stroke or TIA	No	No	No
NIHSS at admission	0	9	0
Stroke localization	Left angular gyrus/inferior parietal lobule	Multiple widespread ischemic lesions, mainly in border zone areas	Multiple widespread ischemic lesions, mainly in border zone areas
Proximal arterial stenosis or occlusion (CTA/MRA)	No	No	No
Acute treatment			
Antithrombotic therapy	Yes	No	Yes
Primary EVT	No	No	No
IVT	No	No	No
IVT + EVT	No	No	No
D-Dimer (ng/mL)	368	2245	>35,000
WBC counts (/mm^3^)	10.77 × 1000	12.32 × 1000	11.7 × 1000
Platelet count (/mm^3^)	153 × 1000	183 × 1000	216 × 1000
CRP (mg/L)	0.09	7.62	2.62
Eosinophils (/mm^3^)	1.64 × 1000	5.54 × 1000	1.28 × 1000
Hypereosinophilia initial treatment	Prednisone 500 mg ev	Prednisone 1 gr ev	Prednisone 1 gr ev
6-months-outcome (mRS scale)	1	4	5

Abbreviations: CPR: C-reactive protein; CTA: Computed Tomography Angiography; EVT: EndoVascular Treatment; IVT: IntraVenous Thrombolysis; MRA: Magnetic Resonance Angiography; NIHSS: National Institute of Health; Stroke Scale; TIA: Transient Ischemic Attack; WBC: white blood cells; mRS: modified Rankin scale.

## Data Availability

Not applicable.
